# Evaluation of the correlation between porphyrin accumulation in cancer cells and functional positions for application as a drug carrier

**DOI:** 10.1038/s41598-021-81725-3

**Published:** 2021-01-21

**Authors:** Koshi Nishida, Toshifumi Tojo, Takeshi Kondo, Makoto Yuasa

**Affiliations:** 1 Department of Pure and Applied Chemistry, Faculty of Science and Technology, Tokyo University of Science, 2641 Yamazaki, Noda, Chiba 278-8510 Japan; 2 Research Institute for Science and Technology, Tokyo University of Science, 2641 Yamazaki, Noda, Chiba 278-8510 Japan

**Keywords:** Chemical biology, Membranes

## Abstract

Porphyrin derivatives accumulate selectively in cancer cells and are can be used as carriers of drugs. Until now, the substituents that bind to porphyrins (mainly at the meso-position) have been actively investigated, but the effect of the functional porphyrin positions (β-, meso-position) on tumor accumulation has not been investigated. Therefore, we investigated the correlation between the functional position of substituents and the accumulation of porphyrins in cancer cells using cancer cells. We found that the meso-derivative showed higher accumulation in cancer cells than the β-derivative, and porphyrins with less bulky substituent actively accumulate in cancer cells. When evaluating the intracellular distribution of porphyrin, we found that porphyrin was internalized by endocytosis and direct membrane permeation. As factors involved in these two permeation mechanisms, we evaluated the affinity between porphyrin-protein (endocytosis) and the permeability to the phospholipid bilayer membrane (direct membrane permeation). We found that the binding position of porphyrin affects the factors involved in the transmembrane permeation mechanisms and impacts the accumulation in cancer cells.

## Introduction

In drug discovery, it is necessary to select a highly biocompatible compound because toxic drugs cannot be clinically used no matter their efficacy^1^. Regarding biocompatibility, porphyrin is an appealing candidate. It is composed of four pyrrole subunits (the 3-position of pyrrole is termed β-position, the methine group that connects pyrroles is termed meso-position) and has 18π-electrons that form a plane. Besides, porphyrin is an active center of the four subunits of hemoglobin, which is the main constituent of human blood. Porphyrin can form conjugates with metals (Fe, Ni, and Co)^2–5^ which provide electrochemical and oxidation–reduction properties. These conjugates can be used as biosensors to determine the amounts of active oxygen in an organism^6,7^. We aim to discover highly versatile and biocompatible porphyrins to reduce the side effects of anticancer agents. Porphyrin can accumulate selectivity in cancer cells^8^. This accumulation is due to π–π stacking between the π-conjugated structure of porphyrin and the aromatic amino acid residues of blood proteins (LDL^9–11^, albumin^12,13^, and transferrin^14,15^) and to the activated endocytosis of these proteins facilitated by receptors activated in cancer cells. Altogether, the accumulation of porphyrin in tumors is caused by a combination of the electronic properties of porphyrin and various receptor activities of cancer cells. Based on this, we aim to produce anti-cancer drugs that have low side effects by combining porphyrin with anticancer drugs^8,16^.

Until now, research on porphyrins as a cancer treatment has focused on their photosensitizing effect^17–19^. Porphyrin photosensitivity can generate anti-cancer activity. Porphyrin is excited by light irradiation, and the energy transferred to oxygen when porphyrin returns to the ground state produces reactive oxygen species that have an anti-cancer activity^20^. Photosensitizers with a porphyrin structure (ex. Photofrin) do not cause damage to normal cells because they are only active when irradiated with light and selectively target tumors thanks to the porphyrin properties. The advantage of tumor accumulation by drug delivery systems (DDS) is the reduction of side effects^21,22^. Anti-cancer drug therapy is an effective cancer treatment. However, side effects caused by toxicity in normal cells are problematic, and DDS that transport drug selectively to cancer cells are of particular importance. As described above, porphyrin can deliver drugs selectively to the affected area. Besides, anti-cancer drugs and porphyrin’s photosensitizing effect can act synergistically to enhance anti-cancer effects^16,23^. Porphyrins have been used as carriers with a wide variety of substituents. Most studies report the addition of substituents on the meso-position (anionic substituents^19,24^, cationic substituents^25,26^, ligand biomolecules for cancer cells receptors^11^, etc.). These studies revealed that the charges of peripheral substituents influence the intracellular distribution, and biomolecule substituents improve the targeting of cancer cells. However, little is known about the influence of these functional positions on porphyrin accumulation in tumors. Here, we focused on β- and meso-positions to elucidate correlations between functional porphyrin positions and accumulation in cancer cells. Understanding this can help define steric effects and lead to the discovery of new molecules with high tumor accumulation.

Based on the above, we aim to elucidate the correlation between the tumor accumulation of porphyrin and the functional position of substituent. We previously demonstrated that the functional position of the substituent (β- or meso-position) greatly affects tumor accumulation and thus must be considered when binding drugs to porphyrins^27^. The affinity between the blood protein and porphyrin at the time of cell membrane permeation must also be considered. Building up from our preceding paper^27^, we evaluated the following three points.

First, we monitored porphyrins accumulation in cancer cells at 2, 6, 16, and 24 h and evaluated the correlation between tumor accumulation and substituent position. Second, we examined the intracellular distribution of porphyrin using a confocal laser scanning microscope to evaluate the pathway through which porphyrin penetrates the cell membrane. Third, we assessed the permeability of porphyrin through the phospholipid bilayer using a model cell membrane “liposome”. By adding the above three points to the previous paper, we aimed to elucidate the mechanism of tumor accumulation change caused by functional position from more various angles. This article is based on a study published in the Bioorganic and Medicinal Chemistry Letters, “Correlations between functional porphyrin positions and accumulation in cancer cells.”

## Results

### Synthesis

**Scheme 1:**
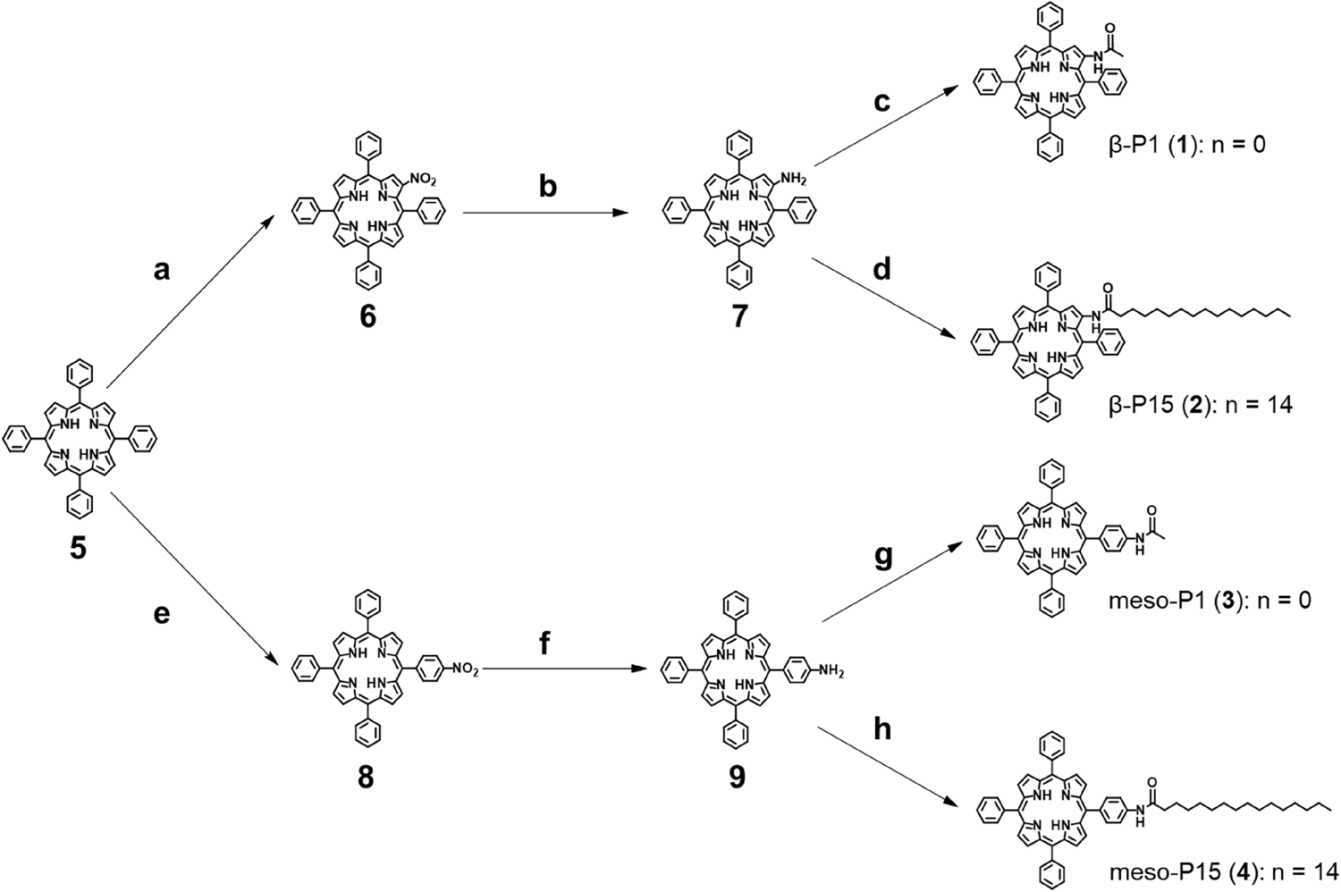
Synthesis of β-P1 (**1**), β-P15 (**2**), *meso*-P1 (**3**) and *meso*-P15 (**4**).Reagents and conditions: (**a**) CH_3_COOH, (CH_3_CO)_2_O, Cu(NO_3_)_2_·3H_2_O, CHCl_3_, r.t., 16 h, then conc. H_2_SO_4_, CH_2_Cl_2_, r.t., 20 min; (**b**) SnCl_2_·2H_2_O, HCl, 65 °C, 5 h, 46% over three steps; (**c**) acetyl chloride, Et_3_N, CH_2_Cl_2_, r.t., 3 h, 34%; (**d**) palmitoyl chloride, Et_3_N, CH_2_Cl_2_, r.t., 3 h, quant.; (**e**) CH_3_COOH, CCl_3_COOH, NaNO_2_, r.t., 2 h; (**f**) SnCl_2_·2H_2_O, HCl, 65 °C, 5 h, 50% over two steps; (**g**) acetyl chloride, Et_3_N, CH_2_Cl_2_, r.t., 3 h, quant.; (**h**) palmitoyl chloride, Et_3_N, CH_2_Cl_2_, r.t., 3 h, 49%.

Scheme 1 shows the synthesis of compounds β-P1 (**1**), β-P15 (**2**), meso-P1 (**3**), and meso-P15 (**4**). First, compound **6** was converted from Tetraphenylporphyrin (H_2_TPP) (**5**) using copper nitrate trihydrate and sulfuric acid^28^. Compound **6** was reduced with SnCl_2_·2H_2_O and hydrochloric acid to yield compound **7**^29^. Compound **7** was condensed with the corresponding carboxylic acid chlorides to provide β-P1 (**1**) and β-P15 (**2**)^30^. Compounds were purified by silica gel chromatography (CHCl_3_). Compound **8** was synthesized by nitration of compound **5** using sodium nitrate^31^ and treated with SnCl_2_·2H_2_O and hydrochloric acid to obtain compound **9**^29^. meso-P1 (**3**) and meso-P15 (**4**) were synthesized using the same conditions as for β-P1 (**1**) and β-P15 (**2**), respectively^30^. β-P1 (**1**), β-P15 (**2**), meso-P1 (**3**), and meso-P15 (**4**) were purified by silica gel chromatography (CHCl_3_) and their identities were confirmed by ^1^H-NMR, ^13^C-NMR, and ESI–MS.

### Porphyrins behavior to MCF-7 cells

**Figure 1:**
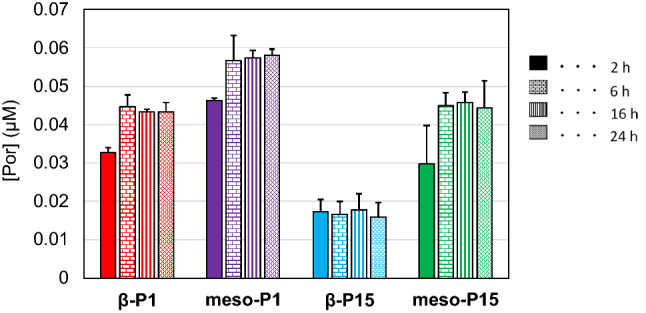
Time-dependent tumor accumulation of porphyrins β-P1 (red), *meso*-P1 (purple), β-P15 (light blue) and *meso*-P1 (green) at 10 µM by MCF-7 cells.

As shown in Fig. 1, β-P1 (**1**), β-P15 (**2**), meso-P1 (**3**), and meso-P15 (**4**) showed remarkably different accumulation behavior in human breast cancer cells (MCF-7). Figure 1 shows the results for each porphyrin’s accumulation amount in MCF-7 cells. Meso-P1 showed the highest accumulation, while β-P15 showed the lowest accumulation regardless of incubation time. The functional position of the substituent and the alkyl chain length both affect the accumulation of porphyrin (meso-P1 > meso-P15, β-P1 > β-P15). Meso-derivatives (meso-P1 and meso-P15) accumulate more than β-derivatives (β-P1 and β-P15) in tumor cells. This was especially obvious in derivatives with a P15 substituent, where the accumulation of meso-derivative was three times higher than β-derivative. Regarding the steric hindrance of the substituent, compounds with a shorter alkyl chain accumulated more than those with a longer alkyl chain. Therefore, a meso-derivative with a bulky functional group may have three times higher anti-cancer effect than a β-derivative.

All compounds reached a maximum accumulation value within six hours, which suggests that the time-dependent performance of porphyrin accumulation in cancer cells is independent of the functional position and substituent.

These results indicated that the accumulation of porphyrin in cancer cells depends on the functional position. We hypothesized that such changes in tumor accumulation might be influenced by factors involved in cell membrane penetration. To investigate where porphyrin accumulates in the cell membrane, we observed the subcellular localization of porphyrin using a time-lapse confocal laser scanning microscope. Porphyrin interacts with plasma proteins to form a complex which is then delivered to intracellular lysosomes by endocytosis^32^. Porphyrins may also accumulate in the cytoplasm by diffusing through the phospholipid bilayer membrane due to a concentration gradient^17^. Accordingly, we investigated how each cell membrane permeation mechanism (endocytosis or concentration gradient) occurs in this experimental system by evaluating the accumulation of porphyrin in lysosomes from fluorescence images.

**Figure 2:**
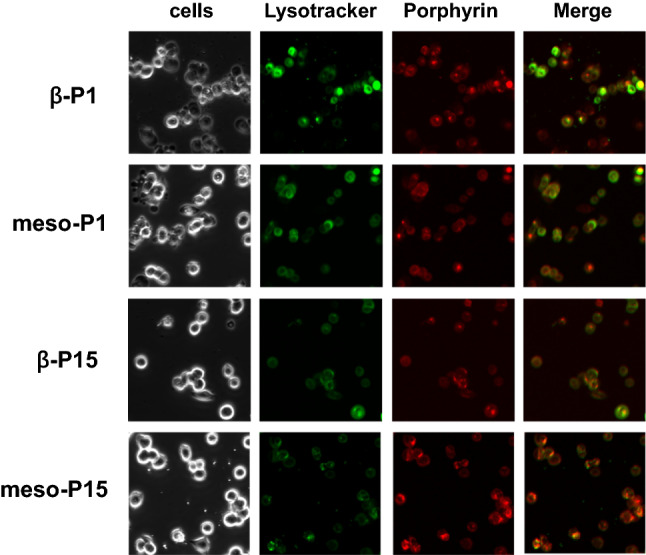
Fluorescence microscope image of test compounds in MCF-7 cells. (Lysotracker and each porphyrin compound are represented by green and red fluorescence, respectively).

Figure 2 shows that each porphyrin derivative was actively delivered to the lysosome by endocytosis. However, the fluorescence images also show that porphyrin derivatives accumulate in other areas than the lysosome. These results indicate that porphyrin derivatives accumulate in cancer cells via both endocytosis and concentration gradient permeation. Therefore, the factors involved in both mechanisms needed to be evaluated and compared for each compound, since they may each have different affinities for blood proteins and different diffusion permeability. Thus, we evaluated and compared these factors among derivatives using fluorescence quenching and a model membrane (liposome).

### Evaluation of transmembrane factors

Meso-derivatives accumulated more than β-derivatives (Fig. 1). Besides, all derivatives were internalized in lysosomes (Fig. 2). We thought that endocytosis of the derivatives into lysosomes might be related to the difference of accumulation. Porphyrin is a π-electron-rich molecule, thus it forms very strong π–π stacking interactions with aromatic amino acid residues of various blood proteins (albumin, LDL, transferrin), and the porphyrin–protein complexes are selectively taken up by cancer cells which overexpressed on the cell membrane surface^8^. Serum albumin is the most abundant protein in the blood (approximately 60%) and it selectively forms a complex with porphyrins by hydrophobic interaction or π–π stacking. Hence, the endocytosis of albumin is the primary pathway for tumor accumulation of porphyrins. Accordingly, we expected that the binding affinity of the porphyrin derivatives for albumin to affect their accumulation in cancer cells. To validate this, we determined binding constants between bovine serum albumin (BSA) and the porphyrin derivatives.

We used the quenching of the fluorescence of BSA to calculate the binding affinity of the porphyrin derivatives for BSA^33^. With this method, binding constants can be calculated using Stern–Volmer plots and Lehrer plots.

**Figure 3:**
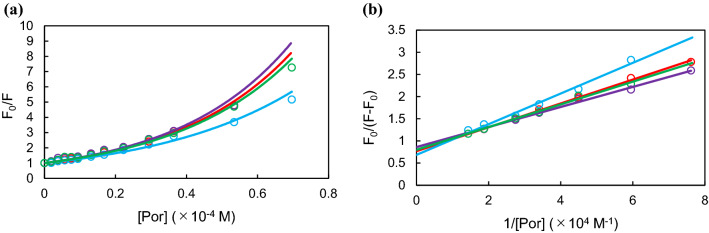
(**a**) Stern–Volmer plots and (**b**) Lehrer plots for the quenching of BSA fluorescence intensity by the test compounds [β-P1(red), meso-P1 (purple), β-P15 (light blue) and meso-P15 (green)]^27^.

Since we proved in our previous paper, it can be proved that porphyrins form a complex with the protein because Stern–Volmer plots show upward curvature in at higher values of quencher concentration (Fig. 3a)^27^. Based on the results of the Lehrer plot (Fig. 3b), the binding constant *K*_a_ between porphyrin and BSA was calculated. The *K*_a_ values were consistent with the accumulation potential of porphyrin derivatives (meso-P1 > meso-P15, β-P1 > β-P15)^27^. These results suggest that π–π stacking between π-conjugated porphyrin derivatives and aromatic amino acid residues of BSA is one of the determining factors of the accumulation into cancer cells.

The results of the fluorescence microscope imagery also revealed that the four porphyrin derivatives localize to the cytoplasm (Fig. 2). Therefore, to elucidate the difference of accumulation between meso-derivatives and β-derivatives, we evaluated the ability of porphyrin derivatives to cross the phospholipid bilayer using liposome as a model of the cell membrane. We used 1,2-dioleoyl-sn-glycero-3-phosphocholine (DOPC) liposome with phosphatidylcholine (PC) polar head and unsaturated chains because the cell membrane is mostly composed of PC and phospholipids with unsaturated chains^34,35^.

Cell membrane permeability was evaluated by quantifying the porphyrin contained in the DOPC liposomes.

**Figure 4:**
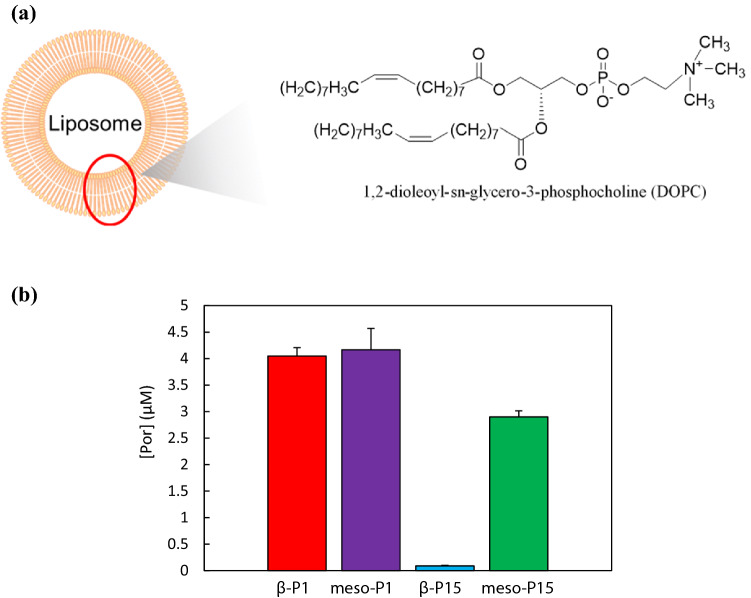
(**a**) Structural formula of DOPC; (**b**) DOPC liposome permeability of the porphyrin derivatives at 10 µM.

As shown in Fig. 4, the cell membrane permeability of porphyrin derivatives greatly depends on the functional position and steric hindrance of the substituents. Meso-P1 and β-P1, bearing a short alkyl chain, showed a high membrane permeation, which did not seem to be affected by the functional position of the substituent. By contrast, Meso-P15 and β-P15, bearing a long alkyl chain, showed lower membrane permeability than the compounds with a short alkyl chain. Besides, β-P15 hardly permeates the DOPC membrane, while meso-P15 does. Thus, changing the functional position of the substituent from the meso-position to the β-position does reduce the cell membrane permeability.

Hence, with these four porphyrins (β-P1, meso-P1, β-P15, meso-P15), we found that modifying the position and steric hindrance of the substituent affects their ability to cross the phospholipid bilayer. First, increasing the steric hindrance of substituents decreases their ability to cross the membrane. This behavior is consistent with the results of the accumulation in MCF-7 cells. Second, β-P15 is hardly incorporated into the phospholipid bilayer membrane. This extremely low permeability of β-P15 may be one of the factors of its extremely low accumulation in MCF-7 cells.

## Discussion

We found that the meso-derivatives showed higher accumulation in cancer cells than the β-derivatives, and the accumulation depended on the steric hindrance of the substituent (Fig. 1). Furthermore, having a bulky substituent at the meso-position resulted in absorption that was three times higher than at the β-position. This indicates that the functional position of the substituent greatly influences drug efficacy, which should be considered when developing a drug delivery system for an anticancer drug with a high steric hindrance.

Next, we attempted to elucidate the effect of functional position on membrane permeation mechanisms. Thus, the membrane permeation process of porphyrins was evaluated using fluorescence microscopy (Fig. 2). From these images, we found that both endocytosis and diffusion are involved in the accumulation of porphyrins MCF-7 cells. Therefore, we considered that the affinity between porphyrin and proteins, and the permeability of phospholipid bilayer membranes to porphyrins are important factors for endocytosis and diffusion, respectively, and evaluated these factors using fluorescence quenching and a model membrane.

The binding constants between the porphyrin derivatives and BSA were determined by fluorescence quenching. As shown in Table 1, the binding constants were consistent with tumor accumulation. This indicates that the porphyrin-protein affinity depends on the functional position of the substituent and this change greatly affects the accumulation in MCF-7 cells. It has been reported that adding a substituent at the β-position increases the strain of the porphyrin ring and reduces the flatness of the porphyrin compound^36^. We suppose that this distorted ring structure decreases the affinity between the porphyrin ring and the aromatic amino acid residues. Next, compounds with a short alkyl substituent showed higher *K*_a_ values than compounds with a long alkyl substituent, regardless of position (meso-P1 > meso-P15, β-P1 > β-P15). The steric effects of bulky substituents also inhibit interactions between porphyrins and aromatic amino acid residues.

**Table 1:** Binding constants (K_α_) between BSA and test compounds.

	K_α_ (L/mol)	Accumulation in MCF-7 (μM)
β-P1	2.86 × 10^4^	4.3 × 10^−2^
Meso-P1	3.81 × 10^4^	5.8 × 10^−2^
β-P15	1.98 × 10^4^	1.7 × 10^−2^
Meso-P15	3.12 × 10^4^	4.4 × 10^−2^

Then, we evaluated the ability of the porphyrin derivatives to diffuse across a phospholipid bilayer using DOPC liposomes as a model of the cell membrane. As shown in Fig. 4, compounds with a long alkyl substituent showed low permeability. Especially the β-derivative (β-P15) showed abnormally low membrane permeability, which reflects its extremely low tumor accumulation. Overall, the membrane permeability depends on the steric hindrance of the substituent and the functional position. There are several reasons for this phenomenon. For example, the inhibition of membrane permeation caused by the bulky substituent, the affinity with the lipid bilayer membrane due to the strain of the porphyrin ring, and the aggregation morphology change in aqueous solution caused by the functional position^37–39^. In particularly, regarding the comparison of the membrane permeability of meso-P15 and β-P15, we expected that the porphyrin aggregation in the aqueous solution affects the membrane permeability. It has been reported that association of porphyrins to liposomes was expected to be a two-steps process: firstly dissociation of the aggregates and secondly, binding or permeating of monomers to liposomes^40^. Therefore, we considered that the aggregation in the aqueous solution is greatly related to the membrane permeability. In addition, the long-alkyl chain substituent of porphyrin increases the hydrophobic interaction between the alkyl chains and increases the self-aggregation^41,42^. Furthermore, the β-position derivative has higher aggregation than the meso-position derivative^43^, and it can be inferred that β-P15, which has a long-alkyl chain and is a β-position derivative, has a structure that promotes aggregation. From the above, it is considered that β-P15 is a most likely to aggregate structure due to the influence of the functional position and the length of the alkyl chain, and it was resulting in a significant decrease in membrane permeability. On the other hand, in the future, the effects of other factors (inhibition of permeation by the bulky substituent, the strain of the porphyrin ring etc.) should be elucidated in more detail.

On the whole, the accumulation of porphyrins greatly depends on the functional position and steric hindrance of the substituent. This interesting phenomenon is caused by the competitive influence of the endocytosis and concentration gradient permeation.

## Conclusion

We investigated the effects of functional positions (β, meso-position) on porphyrins accumulation in cancer cells. We found that the accumulation of the porphyrin derivatives significantly depended on the functional porphyrins position and the substituent’s steric hindrance. We think that such an interesting phenomenon is influenced by the two cell membrane permeation mechanisms of porphyrin, endocytosis, and concentration gradient permeation, and we made great progress in elucidating the mechanism of the effects of functional position substitution by evaluating transmembrane factors. Hence, we found that the functional porphyrin position is crucial when considering the use of porphyrin as a carrier for drug delivery. This study provides new guidelines for the structural design of porphyrin drug products.

## Methods

### General protocol

Reagents and solvents were purchased from Wako pure Chemical Industries and Tokyo Chemical Industry. Column chromatography was performed using silica gel supplied by Fuji Silysia Chemical. Proton nuclear magnetic resonance spectra (^1^H NMR) and carbon nuclear magnetic resonance spectra (^13^C NMR) were recorded using a JEOL JNM-AL300 spectrometer in the indicated solvent. Chemical shifts (δ) are reported in parts per million relative to the internal standard tetramethylsilane. Elemental analyses were recorded on a Perkin Elmer 2400 II CHNS/O analyzer. High-resolution mass spectra (HRMS) were recorded on a JEOL JMS-T100CS mass spectrometer. The Purification of all tested compounds was performed by HPLC using a Japan Analytical Industry LC-9210 with a JAIGEL 1H column (600 mm × φ20 mm, Japan Analytical Industry) (UV detection, λ = 254 nm; flow 1 mL/min; CHCl_3_).

### Synthesis of 2-amino-5,10,15,20-tetraphenylporphyrin (β-H_2_TPAP)^27^

Glacial acetic acid (16 mL), acetic anhydride (100 mL), and copper nitrate trihydrate (1.0 g, 4.26 mmol) were added to a solution of 5,10,15,20-tetraphenylporphyrin (1.0 g, 1.63 mmol) in CHCl_3_ (500 mL). The reaction mixture was stirred at r.t. for 16 h, cooled, and water (200 mL) was added to the mixture. The acetic acid was neutralized with aqueous sodium carbonate. The organic phase was washed with water (200 mL) twice. The organic phase was dried with anhydrous sodium sulfate. The solvent was removed under reduced pressure to give β-CuTPNP, which was used for the next reaction without further purification.

Concentrated H_2_SO_4_ (6.8 mL) was added dropwise to a solution of β-CuTPNP in CH_2_Cl_2_ (350 mL). The mixture was stirred at r.t. for 30 min, water was added (200 mL) and extracted twice. To neutralize sulfuric acid, a saturated solution of sodium carbonate (200 mL) and water (200 mL) were added to the resulting mixture. The organic phase was extracted with CH_2_Cl_2_ and dried over anhydrous sodium sulfate. The solvent was removed under reduced pressure to give β-H_2_TPNP, which was used for the next reaction without further purification.

Tin(II) chloride dihydrate (1.0 g, 4.52 mmol) was added to a solution of β-H_2_TPNP in concentrated hydrochloric acid (50 mL). The mixture was stirred at 65 °C for 5 h under Ar atmosphere, and cooled by adding it to cold water (200 mL). Then, the mixture was adjusted to pH 8 with a saturated solution of sodium carbonate. The organic phase was extracted with chloroform and dried over anhydrous sodium sulfate. The solvent was removed under reduced pressure. The resulting crude solid was purified by chromatography on silica gel (CHCl_3_ only) to give 2-amino-5,10,15,20-tetraphenylporphyrin (466.5 mg, 46% over three steps).

^1^H-NMR (CDCl_3_, 300 MHz, TMS): δ =  − 2.08 (br. s, 2H), 4.42 (br. s, 2H), 7.59–7.84 (m, 13H), 7.88–8.03 (m, 2H), 8.09–8.23 (m, 6H), 8.49–8.63 (m, 3H), 8.67–8.81 (m, 3H) ppm.

### Synthesis of 2-acetyl-5,10,15,20-tetraphenylporphyrin (β-H_2_TPAcP)^27^

Triethylamine (2.4 mL) was added to a solution of β-H_2_TPAP (0.69 g, 1.10 mmol) in CH_2_Cl_2_ (50 mL). After stirring at r.t. for 15 min, acetyl chloride (1.0 mL, 14.1 mmol) was added to the mixture and stirred at r.t. for 3 h. Water (200 mL) was added to the reaction mixture and the organic phase was extracted twice. Saturated sodium hydrogen carbonate (200 mL) and water (200 mL) was added to the resulting mixture. The organic phase was extracted with CHCl_3_, dried over anhydrous sodium sulfate, and the solvent was removed under reduced pressure. The resulting crude solid was purified by chromatography on silica gel (CHCl_3_ only) to give 2-acetyl-5,10,15,20-tetraphenylporphyrin (252 mg, 34%).

^1^H-NMR (CDCl_3_, 300 MHz, TMS): δ =  − 2.83 (br. s, 2H), 1.84 (s, 3H), 7.62–7.94 (m, 13H), 8.13–8.31 (m, 8H), 8.58–8.66 (m, 1H), 8.77–8.90 (m, 5H), 9.31 (s, 1H) ppm. ^13^C-NMR (CDCl3, 75 MHz, TMS): 24.0, 116.1, 119.9, 120.5, 121.1, 126.6, 126.7, 126.8, 127.7, 127.8, 128.3, 129.2, 133.3, 134.3, 134.4, 134.5, 140.8, 141.9, 142.1, 142.3, 167.5 ppm. Anal. Calcd for C_46_H_33_N_5_O: C, 82.24; H, 4.95; N, 10.42%. Found C, 81.15; H, 4.89; N, 9.97%. HRMS (ESI) calcd for C_46_H_33_N_5_O 694.2527 [M+], found 694.2583.

### Synthesis of 2-palmitoyl-5,10,15,20-tetraphenylporphyrin (β-H_2_TPPaP)^27^

Triethylamine (0.83 mL) was added to a solution of β-H_2_TPAP (0.41 g, 0.65 mmol) in CH_2_Cl_2_ (30 mL). After stirring at r.t. for 15 min, palmitoyl chloride (0.66 mL, 2.18 mmol) was added to the mixture and stirred at r.t. for 3 h. Water (200 mL) was added to the reaction mixture and the organic phase was extracted twice. Saturated sodium hydrogen carbonate (200 mL) and water (200 mL) were added to the resulting mixture. The organic phase was extracted with CHCl_3_, dried over anhydrous sodium sulfate, and the solvent was removed under reduced pressure. The resulting crude solid was purified by chromatography on silica gel (CHCl_3_ only) to give 2-palmitoyl-5,10,15,20-tetraphenylporphyrin (775 mg, quant.).

^1^H-NMR (CDCl_3_, 300 MHz, TMS): δ =  − 2.83 (br. s, 2H), 0.88 (t, 3H, *J* = 6.9 Hz, 3H), 1.19–1.34 (m, 24H), 1.48–1.72 (m, 2H), 2.38–2.27 (m, 2H), 7.98–7.95 (m, 13H), 8.15–8.26 (m, 8H), 8.57–8.62 (m, 1H), 8.76–8.56 (m, 5H), 9.36 (s, 1H) ppm. ^13^C-NMR (CDCl_3_, 75 MHz, TMS); 14.1, 22.7, 23.9, 25.5, 29.4, 29.7, 31.9, 37.7, 115.9, 119.9, 120.4, 121.2, 126.6, 126.7, 126.8, 127.7, 127.8, 128.3, 129.2, 133.3, 134.3, 134.4, 134.5, 140.9, 141.9, 142.1, 142.3, 170.5 ppm. Anal. Calcd for: C, 83.01; H, 7.08; N, 8.07%. Found. C, 82.72; H, 6.81; N, 7.83%. HRMS (ESI) calcd for C_60_H_61_N_5_O 890.4773 [M+], found 890.4774.

### Synthesis of 5-(4-Aminophenyl)-10,15,20-triphenylporphyrin (meso-H_2_TPAP)^27^

5,10,15,20-Tetraphenylporphyrin (1.0 g, 1.63 mmol) and NaNO_2_ (0.28 g, 4.06 mmol) were added to a solution of trichloroacetic acid (63.5 g, 388 mmol) in acetic acid (30 mL). The resulting solution was stirred at r.t. for 2 h under Ar atmosphere and water (200 mL) was added to the solution. The mixture was extracted with CHCl_3_. The organic phases were washed with a saturated aqueous NaHCO_3_ solution followed by water. The organic layer was dried over anhydrous sodium sulfate and evaporated under reduced pressure to give meso-H_2_TPNP, which was used for the next reaction without further purification.

Tin(II) chloride dihydrate (1.8 g, 8.00 mmol) was added to a solution of meso-H_2_TPNP in concentrated hydrochloric acid (80 mL). After stirring at 65 °C for 2 h under Ar atmosphere, and the mixture was cooled by adding it to cold water (200 mL). Then, the mixture was adjusted to pH 8 with a saturated solution of sodium carbonate. The organic phase was extracted with chloroform and dried over anhydrous sodium sulfate. The solvent was removed under reduced pressure. The resulting crude solid was purified by chromatography on silica gel (CHCl_3_ only) to give 5-(4-Aminophenyl)-10,15,20-triphenylporphyrin (517 mg, 50% over two steps).

^1^H-NMR (CDCl_3_, 300 MHz, TMS):δ =  − 2.76 (s, 2H), 4.02 (s, 2H), 7.05 (d, *J* = 8.4 Hz, 2H), 7.76 (m, 9H), 8.00 (d, *J* = 8.4 Hz, 2H), 8.22 (m, 6H), 8.76–8.87 (m, 6H), 8.95 (d, *J* = 4.8 Hz, 2H) ppm.

### Synthesis of 5-(4-Acetophenyl)-10,15,20-triphenylporphyrin (meso-H_2_TPAcP)^27^

Triethylamine (3.2 mL) was added to a solution of meso-H_2_TPAP (0.77 g, 1.22 mmol) in CH_2_Cl_2_ (50 mL). After stirring at r.t. for 15 min, acetyl chloride (1.0 mL, 14.1 mmol) was added to the mixture and stirred at r.t. for 3 h. Water (200 mL) was added to the reaction mixture and the organic phase was extracted twice. Saturated sodium hydrogen carbonate (200 mL) and water (200 mL) were added to the resulting mixture. The organic phase was extracted with CHCl_3_, dried over anhydrous sodium sulfate, and the solvent was removed under reduced pressure. The resulting crude solid was purified by chromatography on silica gel (CHCl_3_ only) to give 5-(4-Acetophenyl)-10,15,20-triphenylporphyrin (820 mg, quant.).

^1^H-NMR (CDCl_3_, 300 MHz, TMS):δ =  − 2.85 (s, 2H), 2.22 (s, 3H), 7.60–7.80 (m, 11H), 8.02–8.19 (m, 8H), 8.71–8.83 (m, 8H) ppm. ^13^C-NMR (CDCl_3_, 75 MHz, TMS): 24.5, 117.9, 119.5, 120.1, 126.6, 127.7, 131.0, 134.5, 134.9, 137.7, 137.8, 142.1, 168.7 ppm. Anal. Calcd for: C, 82.24; H, 4.95; N, 10.42%. Found C, 81.69; H, 4.78; N, 9.88%. HRMS (ESI) calcd for C_46_H_33_N_5_O 694.2573 [M+], found 694.2583.

### Synthesis of 5-(4-Palmitoylphenyl)-10,15,20-triphenylporphyrin (meso-H_2_TPPaP)^27^

Triethylamine (1.1 mL) was added to a solution of meso-H_2_TPAP (0.52 g, 0.82 mmol) in CH_2_Cl_2_ (40 mL). After stirring at r.t. for 15 min, palmitoyl chloride (0.83 mL, 2.75 mmol) was added to the mixture and stirred at r.t. for 3 h. Water (200 mL) was added to the reaction mixture and the organic phase was extracted twice. Saturated sodium hydrogen carbonate (200 mL) and water (200 mL) were added to the resulting mixture. The organic phase was extracted with CHCl_3_, dried over anhydrous sodium sulfate, and the solvent was removed under reduced pressure. The resulting crude solid was purified by chromatography on silica gel (CHCl_3_ only) to give 5-(4-palmitoylphenyl)-10,15,20-triphenylporphyrin (350 mg, 49%).

^1^H-NMR (CDCl_3_, 300 MHz, TMS): δ =  − 2.75 (s, 2H), 0.87 (t, *J* = 6.6 Hz, 3H), 1.69–183 (m, 2H), 2.32 (t, *J* = 7.5 Hz, 2H), 1.19–1.45 (s, 24H), 7.57–7.79 (m, 11H), 8.06 (d, *J* = 8.4 Hz, 2H), 8.13–8.25 (m, 6H), 8.78–8.90 (m, 8H) ppm. ^13^C-NMR (CDCl_3_, 75 MHz, TMS): 14.1, 22.7, 25.7, 29.4, 29.5, 29.7, 31.9, 37.9, 117.9, 119.6, 120.2, 126.7, 127.7, 131.2, 134.5, 135.0, 137.6, 138.0, 142.1, 172.1 ppm. Anal. Calcd for: C, 83.01; H, 7.08; N, 8.07%. Found C, 82.39; H, 7.02; N, 7.88%. HRMS (ESI) calcd for C_60_H_61_N_5_O 890.4752 [M+], found 890.4774.

### Cell culture^27^

Human breast cancer (MCF-7) cells, purchased from the Japanese Collection of Research Bioresources (JCRB, Japan), were cultured in 1% (v/v) 100 mM-Sodium Pyruvate Solution (100×) (nacalai tesque), 1% (v/v) 200 mM-l-Glutamine Stock Solution (nacalai tesque), 1% (v/v) Antibiotic–Antimycotic Mixed Stock Solution (100x) (Stabilized) (nacalai tesque) and Dulbecco’s Modified Eagle’s medium (DMEM, nacalai tesque) containing 10% (v/v) fetal bovine serum (FBS, NICHIREI BIOSCIENCES INC).

### Accumulation of porphyrin in MCF-7 cells^27^

MCF-7 cells were cultured in a 6-well plate at initial densities of 500,000 cells/well (2 mL/well) for 24 h (37℃, 5% CO_2_). Subsequently, porphyrin compound solutions (1.0 mM in DMSO) were added (20 μL/well) and incubated for 2, 6, 16, and 24 h.

After incubation, the culture medium was removed, and MCF-7 were washed with PBS three times. SDS buffer (10 wt%) was added to the cells for lysis and the cell lysate was centrifuged (10,000 rpm, 15 min). The fluorescence intensity of the supernatant of the cell lysate was recorded using a JASCO FP-8300 fluorescence spectrometer.

The excitation wavelength was 420 nm. The accumulation concentration of porphyrin in MCF-7 cells was measured by using a calibration curve from the fluorescence intensity.

### Intracellular localization

The MCF-7 cells were seeded onto a glass-bottom dish at initial densities of 50,000 cells/well (2 mL/well) and incubated for 24 h. Lysotracker (Thermo Fisher) was then added to reach a final concentration of 50 nM. Cells were incubated for 2 h under growth conditions. Then the loading solution was replaced with fresh medium and the porphyrin compound was added to reach a final concentration of 10 μM and exposed for 24 h. Intracellular localization of porphyrin was evaluated by using a confocal laser scanning microscope FV-10i. Excitation wavelengths of porphyrins and Lysotracker were 440 nm and 645 nm, respectively.

### Fluorescence quenching measurement^27^

BSA was added to Tris–HCl buffer (50 mM, pH 7.4) containing NaCl (50 mM), at the initial concentration of 9.27 μM. The initial volume of the BSA solution was 2.10 mL. The following volumes of porphyrin stock solution (0.80 mM in DMSO) were added to the BSA solution: P1: 0, 0.005, 0.005, 0.005, 0.005, 0.005, 0.01, 0.01, 0.015, 0.02, 0.02, 0.05 mL, P15: 0, 0.005, 0.005, 0.005, 0.005, 0.005, 0.01, 0.01, 0.015, 0.02, 0.02, 0.05, 0.05 mL. The final volume of the porphyrin stock solution was 0.15 and 0.20 mL; final volume of solution in a cell was 2.25 and 2.30 mL. At each step, the fluorescence intensity of the BSA solution was measured (Excitation wavelength: 420 nm).

The fluorescence intensity characteristic of the aromatic amino acid residues of BSA decreases by adding a porphyrin solution to the BSA solution. Stern–Volmer plots were generated using the Stern–Volmer Eq. (1):

1 $$ F_{0} /F = K_{{sv}} \left[ {{\text{Por}}} \right] + {{1}}  $$

where *F*_*0*_ and *F* are BSA fluorescence intensities in the absence and presence of porphyrin, respectively; *K*_*sv*_ is the Stern–Volmer fluorescence quenching constant; [Por] is the concentration of porphyrin.

The Stern–Volmer Eq. (1) was incorporated into the Lehrer Eq. (2):

2 $$ F_{0} /\left( {F_{0} {-}F} \right) = {1}/\left( {\alpha K_{a} \left[ {{\text{Por}}} \right]} \right) + {1}/\alpha $$

where *F*_0_ and *F* are the BSA fluorescence intensity in the absence and presence of quencher (Por), respectively, [Por] is the concentration of quencher, *K*_*a*_ is the binding constant and *α* is the BSA fraction of the accessible to the quencher fluorophore population.

### Porphyrins incorporation into liposomes

Lipid films containing 1,2-dioleoyl-sn-glycero-3-phosphocholine (DOPC) (43.4 mg, 50 μmol) were prepared by the evaporation of chloroform solutions in glass tubes. The suspension was hydrated with 2 mL of PBS and vortexed for 5 min. The mixture was centrifuged for 5 min at 15,000 rpm, 25 °C, and the supernatant (containing smaller vesicles) was discarded. An equivalent volume of buffer solution was added, and the pellet was re-suspended. This procedure was repeated at least twice to prepare multi-lamellar vesicles (MLV).

To liposome suspension (2 mL) was added to the porphyrin solution (20 μL, 1.0 mM in DMSO) and incubated for 1 h. Then the MLV suspension was centrifuged for 15 min at 15,000 rpm, and the supernatant was removed and 10 wt% SDS buffer (1 mL,) was added to the sediment to destroy the membrane. The fluorescence intensity of porphyrin in the solution was measured using a JASCO FP-8300 fluorescence spectrometer.

## Data Availability

The data that support the findings of this paper are available from the corresponding author, T.T., upon reasonable request.

## References

[1] Zhang Y, Lovell JF (2012). Porphyrins as theranostic agents from prehistoric to modern times. Theranostics.

[2] Ali H, van Lier JE (1999). Metal complexes as photo- and radiosensitizers. Chem. Rev..

[3] Biesaga M, Pyrzynska K, Trojanowicz M (2000). Supramolecular chemistry of metalloporphyrins. Talanta.

[4] Beletskaya I, Tyurin VS, Yu A, Guilard R, Stern C (2009). Supramolecular chemistry of metalloporphyrins. Chem. Rev..

[5] Hambright P (1971). The coordination chemistry of metalloporphyrins. Coord. Chem. Rev..

[6] Yuasa M (2010). Preparation of manganese porphyrin complex-introduced niosomes and evaluation of antioxidant activity. Kobunshi Ronbunshu.

[7] Yuasa M, Oyaizu K, Murata H, Toyoda Y, Namba M, Shitara M (2008). Synthesis of 6-coordinated proximal base bonded iron porphyrin complex and evaluation as an active oxygen sensor. Kobunshi Ronbunshu.

[8] Josefsen L, Boyle RW (2012). Unique diagnostic and therapeutic roles of porphyrins and phthalocyanines in photodynamic therapy, imaging and theranostics. Theranostics.

[9] Dozzo P, Koo MS, Berger S, Forte TM, Kahl SB (2005). Synthesis, characterization, and plasma lipoprotein association of a nucleus-targeted boronated porphyrin. J. Med. Chem..

[10] Jori G (1984). Evidence for a major role of plasma lipoproteins as hematoporphyrin carriers in vivo. Cancer Lett..

[11] Kessel D (1986). Porphyrin-lipoprotein association as a factor in porphyrin localization. Cancer Lett..

[12] Hamblin MR, Newman EL (1994). Photosensitizer targeting in photodynamic therapy I. Conjugates of haematoporphyrin with albumin and transferrin. J. Photochem. Photobiol. B..

[13] Brasseur N, Langlois R, La Madeleine C, Ouellet R, van Lier JE (1999). Receptor-mediated targeting of phthalocyanines to macrophages via covalent coupling to native or maleylated bovine serum albumin. Photochem. Photobiol..

[14] Ward JH, Jordan I, Kushner JP, Kaplan J (1984). Hemin, chelatable iron, and the regulation of transferrin receptor biosynthesis. J. Biol. Chem..

[15] Gijsens A (2002). Targeting of the photocytotoxic compound AlPcS4 to hela cells by transferrin conjugated peg-liposomes. Int. J. Cancer..

[16] Ol’shevskaya VA (2006). Novel boronated derivatives of 5,10,15,20-tetraphenylporphyrin: Synthesis and toxicity for drug-resistant tumor cells. Bioorg. Med. Chem..

[17] Tsubone TM, Martins WK, Pavani C, Junqueira HC, Itri R, Baptista MS (2017). Enhanced efficiency of cell death by lysosome-specific photodamage. Sci. Rep..

[18] Bonnett R (1995). Photosensitizers of the porphyrin and phthalocyanine series for photodynamic therapy. Chem. Soc. Rev..

[19] Hiriuchi H (2011). Silylation enhancement of photodynamic activity of tetraphenylporphyrin derivative. J. Photochem. Photobiol. A..

[20] Abrahamse H, Hamblin MR (2016). New photosensitizers for photodynamic therapy. Biochem. J..

[21] Luderer MJ, de la Puente P, Azab AK (2015). Advancements in tumor targeting strategies for boron neutron capture therapy. Pharm. Res..

[22] Song R, Kim Y-S, Sohn YS (2002). Synthesis and selective tumor targeting properties of water soluble porphyrin–Pt(II) conjugates. J. Inorg. Biochem..

[23] Kralov J (2010). Porphyrin−cyclodextrin conjugates as a nanosystem for versatile drug delivery and multimodal cancer therapy. J. Med. Chem..

[24] Gelfuso GM, Figueiredo FV, Gratieri T, Lopez RFV (2008). The effects of pH and ionic strength on topical delivery of a negatively charged porphyrin (TPPS4). J. Pharm. Sci..

[25] Couto GK (2020). Tetra-cationic platinum(II) porphyrins like a candidate photosensitizers to bind, selective and drug delivery for metastatic melanoma. J. Photochem. Photobiol. B..

[26] Lei W, Jiang G, Zhou Q, Zhang B, Wang W (2010). Greatly enhanced binding of a cationic porphyrin towards bovine serum albumin by cucurbit[8]uril. Phys. Chem. Chem. Phys..

[27] Tojo T, Nishida K, Kondo T, Yuasa M (2020). Correlations between functional porphyrin positions and accumulation in cancer cells. Bioorg. Med. Chem. Lett..

[28] Abdulaeve IA (2016). On the synthesis of functionalized porphyrins and porphyrin conjugates via β-aminoporphyrins. New J. Chem..

[29] Kruper WJ, Chamberlin TA, Kochanny M (1989). Regiospecific aryl nitration of meso-substituted tetraarylporphyrins: A simple route to bifunctional porphyrins. J. Org. Chem..

[30] Coliman JP, Gagne RR, Reed CA, Halbert TR, Lang G, Robinson WT (1975). Picket fence porphyrins. Synthetic models for oxygen binding hemoproteins. J. Am. Chem. Soc..

[31] Bhatt RK, Sharma S, Nath M (2012). La(OTf)_3_-catalyzed one-pot synthesis of meso-substituted porphyrinic thiazolidinones. Monatsh. Chem..

[32] Sharman WM, Allen CM, van Lier JE (1999). Photodynamic therapeutics: Basic principles and clinical applications. Drug Disc. Today..

[33] Makarska-Bialokoz M (2018). Interactions of hemin with bovine serum albumin and human hemoglobin: A fluorescence quenching study. Spectrochim. Acta A. Mol. Biomol. Spectrosc..

[34] Attwood SJ, Choi Y, Leonenko Z (2013). Preparation of DOPC and DPPC supported planar lipid bilayers for atomic force microscopy and atomic force spectroscopy. Int. J. Mol. Sci..

[35] Kaneshina S, Ichimori H, Hata T, Matsuki H (1998). Barotropic phase transitions of dioleoylphosphatidylcholine and stearoyl-oleoylphosphatidylcholine bilayer membranes. Biochim. Biophys. Acta..

[36] Senge MO, Kalisch WW (1997). Synthesis and structural characterization of nonplanar tetraphenylporphyrins and their metal complexes with graded degrees of β-ethyl substitution. Inorg. Chem..

[37] Lang K, Mosinger J, Wagnerová DM (2004). Photophysical properties of porphyrinoid sensitizers non-covalently bound to host molecules; models for photodynamic therapy. Coord. Chem. Rev..

[38] Shelnutt JA, Song X-Z, Ma J-G, Jia S-L, Jentzena W, Medforth CJ (1998). Nonplanar porphyrins and their significance in proteins. Chem. Soc. Rev..

[39] Song X (1996). Langmuir–Blodgett films of stearic acid containing octakis((methoxycarbonyl)methyl)-mesotetrakis(((eicosanyloxy)carbonyl)phenyl)porphyrin. Langmuir.

[40] Hanadi I, Athena K, Changjiang Y (2011). *Meso*-tetraphenyl porphyrin derivatives: The effect of structural modifications onbinding to DMPC liposomes and albumin. J. Photochem. Photobiol. A Chem..

[41] Mamardashvili GM, Mamardashvili NZ, Golubchicov OA, Berezin BD (2001). The influence of alkyl bridge substitution on the porphyrin solubility. J. Mol. Liq..

[42] Mamardashvili GM, Mamardashvili NZ, Berezin BD (2000). Solubility of alkylporphyrins. Molecules.

[43] Ladomenou K, Kitsopoulos TN (2014). The importance of various anchoring groups attached on porphyrins as potential dyes for DSSC applications. RSC Adv..

